# Constructing a “periodic table” of bacteria to map diversity in trait space

**DOI:** 10.1093/ismejo/wraf289

**Published:** 2026-01-01

**Authors:** Michael Hoffert, Evan Gorman, Manuel E Lladser, Noah Fierer

**Affiliations:** Department of Ecology and Evolutionary Biology, University of Colorado Boulder, Boulder, CO 80309, United States; Cooperative Institute for Research in Environmental Sciences, University of Colorado Boulder, Boulder, CO 80309, United States; Department of Applied Mathematics, University of Colorado Boulder, Boulder, CO 80309, United States; Department of Applied Mathematics, University of Colorado Boulder, Boulder, CO 80309, United States; Department of Ecology and Evolutionary Biology, University of Colorado Boulder, Boulder, CO 80309, United States; Cooperative Institute for Research in Environmental Sciences, University of Colorado Boulder, Boulder, CO 80309, United States

**Keywords:** microbial ecology, genomics, taxonomy, bacteria, bioinformatics, periodic table, phylogenetics

## Abstract

Despite an ever-expanding number of bacterial taxa being discovered, many of these taxa remain uncharacterized with unknown traits and environmental preferences. This diversity makes it challenging to interpret ecological patterns in microbiomes and understand why individual taxa, or assemblages, may vary across space and time. Although we can use information from the rapidly growing databases of bacterial genomes to infer traits, we still need an approach to organize what we know, or think we know, about bacterial taxa to match taxonomic and phylogenetic information to trait inferences. Inspired by the periodic table of the elements, we have constructed a “periodic table” of bacterial taxa to organize and visualize monophyletic groups of bacteria based on the distributions of key traits predicted from genomic data. By analyzing 50 745 genomes across 31 bacterial phyla, we used the Haar-like wavelet transformation, a model-free transformation of trait data, to identify clades of bacteria which are nearly uniform with respect to six selected traits*—*oxygen tolerance, autotrophy, chlorophototrophy, maximum potential growth rate, GC content, and genome size. The identified functionally uniform clades of bacteria are presented in a concise periodic table-like format to facilitate identification and exploration of bacterial lineages in trait space. While our approach could be improved and expanded in the future, we demonstrate its utility for integrating phylogenetic information with genome-derived trait values to improve our understanding of the bacterial diversity found in environmental and host-associated microbiomes.

## Introduction

The periodic table of elements offers an interpretable framework for organizing atomic elements based on their properties [1]. This framework has been adapted across various fields to categorize the equivalent of elements, from food types to cell types, according to shared properties [2–5]. In fields like microbial ecology where organisms are the subjects of study, microbial taxa are documented in databases like the Genome Taxonomy Database [6] and BacDive [7] which serve to organize reference genomes, phylogenetic trees, and trait information. However, these databases omit a key feature of a periodic table: organizing units according to functional information. Microbial traits are as much the central focus of microbial ecology as chemical properties are the focus of physical chemistry: traits of microbes are analogous to chemical properties of atoms, where each determine the outcomes of small-scale interactions in their respective systems. Therefore, building a periodic table of microbial diversity that clearly organizes taxa and lineages based on shared traits would highlight key differences and illustrate patterns or periodicity in what we know, or think we know, about the diversity of bacterial life on Earth. However, unlike the clearly periodic composition and configuration of subatomic particles in atoms, bacterial traits are continuous, innumerable, and complexly distributed. Although the functional attributes of microbial taxa and the linkages between these attributes and ecological processes is an active area of research [8–12], there is no consensus about how to interpretably measure, organize, or visualize the distributions of microbial traits across large swaths of taxonomic diversity. Establishing quantitative frameworks that link bacterial taxonomic identity with trait distributions would not only facilitate meaningful comparisons and insights across the rapidly expanding landscape of microbial research, but could also serve as a quantitative means to delineate ecological and functional guilds of bacteria.

Despite its promise, constructing a “periodic table of bacterial taxa” (PTBT) which succinctly visualizes bacterial trait-taxonomy relationships is challenging. Finding and assigning trait measurements to taxa in the PTBT is hampered by the intrinsic difficulty of measuring most bacterial traits, the presence of nebulous or dated taxonomic assignments [13, 14], complex trait distributions arising from horizontal gene transfer [15], and variable rates of evolution [16]. Although commonly cited examples of broad taxonomic groups with conserved metabolisms exist, such as photoautotrophic *Cyanobacteria* [17], most bacterial traits are “distributed in phylogenetic clusters with a continuum of depths” [18] and cannot be uniformly attributed to a taxonomic group without empirical evidence. Establishing such empirical estimates of trait values for taxonomic groups is an evolving challenge. The traits of many bacterial taxa remain unknown because they are resistant to cultivation and not readily amenable to experimentation. However, cultivation-independent methods which infer trait values from genomic data [19] promise to circumvent these challenges and make it feasible to infer some traits from genomic data alone. As ongoing sequencing efforts yield thousands of new bacterial genomes per year, a combination of cultivation-independent and -dependent methods have yielded databases of bacterial traits for an increasing diversity of bacteria [7, 20, 21]. A wide range of bacterial traits can now be inferred from genomic information, including autotrophy [22, 23], nitrogen fixation [24], antibiotic resistance [25], maximum potential growth rates [26], temperature tolerances [27–29], pH preferences [30], and oxygen tolerances [31]. These advances have provided new means by which traits can be inferred for uncultivated and cultivated taxa alike, providing the empirical estimates of traits needed to build a PTBT.

**Figure 1:**
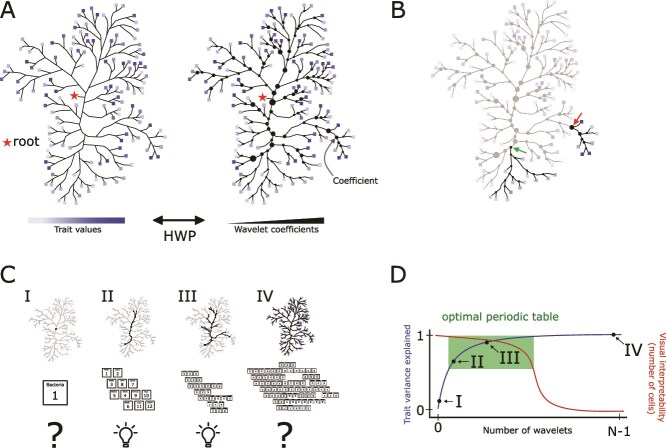
Conceptual outline of our method for constructing a periodic table of bacterial taxa (PTBT). The goal of a PTBT is to describe the distribution of traits across bacterial taxa for phylogenies with hundreds of thousands of leaves using quantitative methods. (A) We first apply the Haar-like wavelet projection (HWP) to a large, complex phylogeny with a trait measured for each leaf, e.g. a trait value predicted from a bacterial genome. The resulting Haar-like wavelet coefficients are associated with internal nodes of the phylogeny and measure the amount of variance uniquely associated with each node. (B) Our method of constructing a PTBT identifies the ancestral nodes of clades with small wavelet coefficients (green arrow), which are monophyletic groups with relatively uniform trait values. In contrast, clades with large wavelets (red arrow) cannot be accurately summarized by representing the collection of leaves with their average trait values. (C) The PTBT is constructed by collapsing clades with minimal variance in the trait to their ancestral node and using the tips of the collapsed phylogeny as “cells” in the table. The degree of collapsing is continuous: complete collapsing of the phylogeny (resulting in a single cell, tree I) is undesirable, as is the original, complex phylogeny (tree IV). An intermediate representation (trees II and III) illustrates which clades are functionally variable or uniform in a visually interpretable number of cells. (D) Therefore, an optimal PTBT selects the largest wavelets that adequately summarize trait-taxon relationships (green box) in addition to preserving visual interpretability.

Here we show how a PTBT can be constructed using recently developed approaches to identify and visualize phylogenetic groups with particular trait distributions. Our approach addresses the challenges of finding trait information for most bacteria and interpreting the complex distribution of those traits in large phylogenies in two parts; first using genome-derived trait estimates to characterize a large swath of bacterial phylogenetic diversity and then applying the recently developed Haar-like wavelet projection (HWP) [32] to identify phylogenetic groups with conserved traits (Fig. 1). The example presented here focuses on six key traits (genome size, GC content, oxygen tolerance, autotrophy, chlorophototrophy, and maximum potential growth rate), traits which can be accurately estimated from genomic data and capture broad gradients in energetics, redox states, carbon sources, and life history strategies. We estimate each trait for over 50 000 representative genomes in the Genome Taxonomy Database (GTDB) [16]. We use the HWP to compute the trait variance associated with each phylogenetic node and identify clades with minimal trait variation—clades which can be accurately represented in single cells in a prototype periodic table (Fig. 1A and B). Because many traits exhibit some degree of phylogenetic conservation [18, 33], we expect that a small number of phylogenetic splits can capture large amounts of trait variance, and identification of these splits via HWP will make it feasible to design a PTBT simple enough to link taxa to traits and accurately describe most of the variance in these traits (Fig. 1C and D). Our approach represents an interpretable and quantifiable method to systematically characterize large components of trait variance at the scale of the bacterial tree of life. We hope that our use of a quantitative means to discover and arrange the cells of the PTBT will allow this method to be readily expanded to add additional traits that are currently challenging to predict from genomic data alone, tailored to assess particular hypotheses about trait-taxonomy relationships, and widened to include additional taxa as genome-based models, experimental training data, and genomic datasets continue to improve.

## Materials and methods

### Compilation of genomic data

62 291 representative genomes from GTDB release 207 (https://data.gtdb.ecogenomic.org/releases/release207/207.0/) were downloaded for annotation with six traits, as described below. We restricted our analyses only to those genomes from phyla with 100 or more representatives. To ensure that maximum estimated growth rate could be included for all genomes, only those containing at least one ribosomal protein were included in the analyses. The remaining 50 745 genomes were used for subsequent analyses. Analyses and figures were generated using Python v3.10.10. Details on the 31 phyla represented and the number of genomes per phylum are provided in Supplementary Fig. 1.

### GC content and genome size

GC content and genome size were drawn directly from GTDB genome statistics, as calculated by CheckM [34].

### Oxygen tolerance

A list of NCBI taxon IDs in BacDive (accessed 6/9/2022) labeled as either “aerobe” or “anaerobe” were used to construct an oxygen tolerance dataset. Seven original BacDive oxygen tolerance labels from 6629 genomes were grouped into two categories using the following scheme: anaerobe (22.2%), obligate anaerobe (1.5%), microaerophile (10.6%), facultative aerobe (0.8%), aerotolerant (0.08%), and microaerotolerant (0.03%) were assigned to anaerobe. Aerobe (46.1%), obligate aerobe (2.2%), and facultative anaerobe (8.2%) were assigned to aerobe. Each NCBI Taxon ID was matched to a representative GenBank assembly and corresponding GTDB representative genome. If no GenBank assembly was labeled as representative, we selected the highest-quality genome based on GTDB’s contamination and completeness estimates and used its GTDB species representative. When GTDB species clusters contained multiple oxygen annotations, the most common label for each cluster was paired to the respective genome. Statistics of the assembled dataset are available in the code resources (see Data Availability). 6629 genomes with labels were identified, 662 used in final testing and 5964 in cross-validation procedures.

A random forest model implemented in sklearn v1.0.2 was used to predict oxygen tolerance. Protein families from Pfam present in each genome were used as predictors with oxygen tolerance categories from BacDive [7]) used as the target variable. The predicted coding sequences from representative genomes were annotated using hmmscan (HMMER v.3.3.2) and PFam release 35.0 (pfam.xfam.org) on open reading frames (ORFs) identified with Prodigal v2.6.3 [35], filtering to hits with bitscores better less than the respective Pfam entry’s “trusted cutoff.” After all matching Pfam families per gene were determined, a presence/absence table was constructed with all 62 291 genomes in release 207 (later filtered to 50 745, as above) and 17 422 (out of 19 632) observed Pfam entries. A bootstrapped logistic regression with an L1 optimizer implemented in sklearn v1.0.2 [36] was used with 100 replicates and lambda = 1 to reduce the number of genes in the input data. The fraction of bootstraps with non-zero regression coefficients for each gene was included in later hyperparameter tuning for random forests.

We withheld 10% of data for model testing (herafter “testing data”) and retained 90% (5964) of the 6629 genomes in the training set for hyperparameter tuning using nested cross-validation, with 10-fold cross-validation for models and five-fold cross-validation for combinations of parameters. We tuned the following parameters: (i) number of trees, (ii) maximum tree depth, (iii) minimum samples per leaf, (iv) minimum samples per split, (v) bootstrapping, (vi) number of features used to train each tree, and (vii) number of bootstrapped logistic L1 regressions with a nonzero coefficient. Maximum training accuracy was achieved using Pfam entries with non-zero coefficients in 50%–70% of randomized logistic regressions and more than 75 trees, but otherwise models were not sensitive to parameter choice. The final model used the following parameters to reduce risk of overfitting: Pfam entries present in 70% of bootstrapped logistic regressions, n_estimators = 3000, max_features = sqrt, max_depth = 13, min_samples_split = 2, min_samples_leaf = 1, bootstrap = False. The random forest model achieved 92% cross-validation training accuracy and 91.4% test accuracy with modest differences in performance for aerobes and anaerobes (Supplementary Fig. 2), likely due to unbalanced training data and better predictive power for aerobe-associated enzymes [31]. The model successfully recapitulated the original BacDive labels in the classification probability space (Supplementary Fig. 3) and used enzymes associated with oxygen-dependent and independent metabolisms (Supplementary Fig. 4).

### Inferring chlorophototrophy

Due to difficulties distinguishing rhodopsin from bacteriorhodopsin, we have focused on chlorophototrophic organisms in this study. To identify proteins and pathways which indicate phototrophy, we identified the following chlorophototrophy-related GO terms: GO:0015995, GO:1902326, GO:0036068, GO:00333005, GO:0030494, GO:0010380, GO:1902325, GO:0036067, GO:0015979, GO:0019684, GO:0019685, GO:0010109, GO:1905157, GO:0009521, GO:1905156, GO:0034357. These GO terms were used to identify 48 Pfam families of photosynthesis-related proteins. The GenBank assembly accessions for known chlorophototrophs from [37] were used to identify which phototrophy-related Pfam families were common among phototrophic taxa. Among 698 GTDB representative genomes identified as phototrophs, 24 had none of the Pfam families. The Pfams for Photosynthetic reaction center protein (PF00124), magnesium-protoporphyrin IX methyltansferase (PF07109), and proto-chlorophyllide reductase (PF08369) were the most prevalent, indicating the bacteriochlorophyll biosynthetic mechanism can be used to identify putative phototrophs. 95.7% of the 698 genomes in [37] contained at least one complete (100% GapSeq completeness) biosynthetic process for chlorophyll a. Genomes were pre-screened using photosynthesis-related Pfam families: 12906 of the 62 291 GTDB representative contained at least one. These 12 906 genomes were scanned with Gapseq v1.2 [38] for chlorophyll photosynthesis capacity. Any genome with at least one chlorophyll-biosynthesis-related process that was more than 90% complete was considered phototrophic for downstream analyses.

### Inferring carbon fixation

Carbon fixation potential was determined by analyzing genomes for genes associated with known carbon fixation pathways from previous literature and existing databases [39–41]. The methods from [23] were used to annotate capacity for autotrophy in GTDB. To initially screen genomes for high pathway completeness of oxidative variants of cycles like the reductive tricarboxylic acid (rTCA) cycle, the HMMs from [23] were run against each genome. After pre-screening, 10 166 genomes were analyzed with GapSeq to measure pathway completeness of the following MetaCyc pathways: CALVIN-PWY / Calvin Cycle, P23-PWY / rTCA I, PWY-5392 / rTCA II, PWY-5789 / 3HP/4HB, PWY-5743 / 3HP bicycle. We used the pathways and pathway completeness thresholds from [23] to identify putative autotrophs, which were determined by measuring the completeness of each carbon fixation pathway in well-studied model organisms. With most reactions containing at least 10 enzymes, it is unlikely that non-autotrophic taxa contain both homologs of key enzymes and very high degrees of completeness of these pathways. Using these cutoffs, 6511 genomes out of 50 745 were identified with sufficient completeness of carbon fixation initially; only the 4627 genomes containing Calvin Cycle or 3HP bi-cycle were considered autotrophic for the construction of the periodic table because other pathways were found in organisms not reported in the literature to be capable of carbon fixation.

### Maximum potential growth rate estimates

To estimate maximum predicted doubling times for each genome, ribosomal proteins were annotated and processed using gRodon v2 [42], a codon usage bias (CUB) based method for estimating microbial doubling time. Ribosomal proteins were used as the highly expressed gene set (from which gRodon estimates growth rates) for each genome. To annotate ribosomal proteins, we used BLASTP v2.5.0 [43] to align predicted ORFs against the growthpred database of microbial ribosomal proteins [44]. ORFs with at least 50% coverage and an e-value of 1e-5 were labeled as ribosomal. 3053 genomes had no hits. Of the remaining 50 771 genomes, 29 642 had fewer than 10 annotated ribosomal genes (Supplementary Fig. 5) and taxa with lower growth rates were biased toward isolate (vs. metagenome-assembled genome) sources (Supplementary Fig. 6).

### Wavelet projection and construction of the periodic table

All subsequent analyses and figures were generated using Python v3.10.10 and ETE toolkit v3.1.2 (https://github.com/etetoolkit/ete). The GTDB r207 phylogeny was pruned using the ete3 “prune” function with keep_branch_lengths = True to contain quality-controlled species with estimates for all six traits (50 745 total). A Haar-like wavelet basis was computed for this tree with code from [32] (https://github.com/edgor17/Sparsify_Ultrametric) and used to compute a 50 744 × 6 matrix of Harr-like wavelets coefficients per trait for each non-root interior node. The vector of Haar-like wavelet coefficients for each trait were divided by the L2 norm, yielding a normalized vector of variances explained per wavelet coefficient/ internal node. To identify nodes which would define a periodic table composed of functionally uniform groups, the normalized wavelet coefficients were sorted and cumulatively summed starting with the largest wavelet until a selected amount of variance was captured (60%) for each trait. Values for six random traits were simulated 100 times under a Brownian motion model with σ^2^ = 1 were used to confirm that large wavelets co-occurred at particular nodes more frequently than expected at random. Subtrees that did not contain any of the 72 total nodes identified by this procedure were collapsed using a custom tree-pruning algorithm.

Algorithm to construct a collapsed tree *T_W_:*

(1) Define a set of nodes *W* to avoid collapsing
(2) Begin with the original phylogeny *T*
(3) Traverse *T* Starting with the root of *T*;

For node *N* in *T:*

- (a) If *N* is a leaf of *T*:
  i) mark it as a leaf of *T_W_*.
- (b) Else if all descendants of *N* are not in *W*:
  i) mark *N* as a leaf of *T_W_*.
- (c) Else:
  i) Continue

(4) Build *T_W_* using only marked nodes as leaves; collapse all unmarked subtrees.

This algorithm retains nodes only if they belonged to a wavelet clade or were necessary to maintain the tree structure connecting retained nodes. This algorithm pruned the original 50 745 tips to 272 tips (clades from the original phylogeny). Re-computing the trait data from the wavelet coefficients in this pruned tree would create a trait distribution that captured 60% or more of the variation from the original data.

These 272 clades were used to draw the periodic table. A 272 × 272 patristic distance matrix of these clades and a 272 × 6 matrix of the scaled median trait values for the leaves associated with each clade was used to compute a three-dimensional ENS-t-SNE embedding with 4000 iterations and a perplexity of 50 using mview (https://github.com/enggiqbal/MPSE-TSNE) [45]. The images from the resulting embedding were combined and points were snapped to a grid using the Hungarian algorithm implemented in sklearn. The grid layout, median trait values for each clade, and phylogenetic tree were used to generate the periodic table diagram and tree diagrams in Fig. 4 and 5 using custom Python scripts and the seaborn [46], numpy [47], and matplotlib [48] libraries.

## Results and Discussion

### Compilation of trait data for 50 745 bacterial genomes

To accomplish the central task of arranging bacterial taxonomic in trait space, we first downloaded 62 291 species-level representative genomes from the GTDB version 207 [6] for trait estimation. We used representative genomes only to ensure most genomes were relatively complete, uncontaminated, and had a standardized taxonomic assignment aligned with the GTDB phylogenetic tree. Our analyses were restricted to the 31 bacterial phyla which included at least 100 species-level representative genomes per phylum (Supplementary Fig. 1) and only genomes with at least one ribosomal protein, a requirement for growth rate predictions [26], yielding 50 745 of the original 62 291 bacterial genomes. For each of these 50 745 genomes, we inferred values for six ecologically relevant traits: oxygen tolerance, autotrophy, chlorophototrophy, maximum potential growth rate, GC content, and genome size as described below and in the Methods section which includes full details on how these traits were determined from the genomic data. Supplementary Table 1 includes the inferred trait values for all 50 745 genomes. We emphasize that our trait inferences, particularly the inferences of autotrophy, phototrophy, and O_2_ tolerance, are approximations which do not include all possible pathways for these functions and could ultimately be improved. However, as detailed below, our inferences broadly align with published literature, despite the literature’s bias toward taxa that are readily cultured and studied in vitro. Likewise, we describe the general patterns at broad taxonomic levels, acknowledging that the phylogenetic depth at which taxa exhibit consistency in traits varies depending on the trait and lineage in question (as discussed in more detail in the following sections).

**Figure 2:**
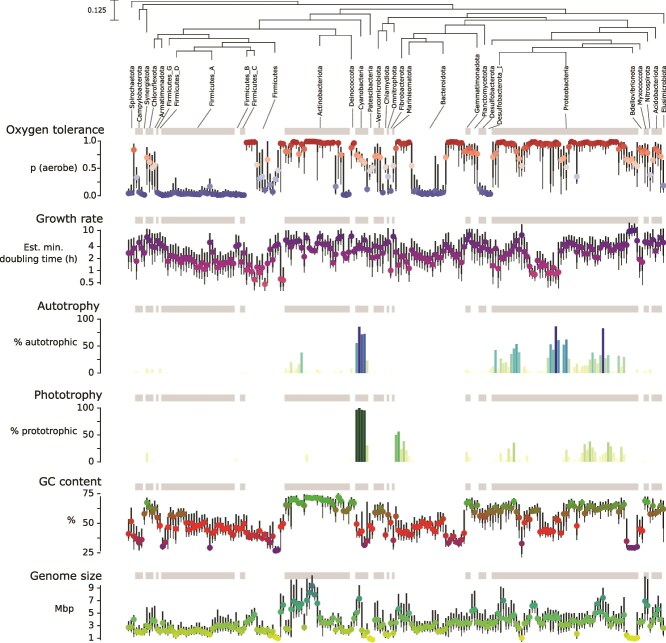
Distributions of estimated trait values for 50 745 GTDB representative genomes for six key traits. To ensure the data from phyla with small numbers of genomes are visible on this plot, the data are collapsed into bins represented by vertical boxplots with colored medians: each phylum is represented by at least one boxplot, where each boxplot shows a color-coded median and the 10th, 25th, 75th, and 90th quantile values for each trait across up to 500 genomes. The gray bands at the top of each plot act as a visual aid to delimit the boxplots corresponding to each phylum, with larger phyla occupying a larger number of boxplots, identified by larger white or gray bands. The phylogenetic tree is drawn using the phylogenetic placement and branch length scale of the 31 included bacterial phyla from the genome taxonomy database (GTDB) v207. Because autotrophy and phototrophy are binary variables, they are shown as bars representing the fraction of genomes in each bin that are positive for that trait. The six measured traits are shown in the following order: (i) oxygen tolerance, the probably of being aerobic, predicted using a random forest trained on BacDive genomes (ii) maximum potential growth rate in units of estimated minimal doubling time from gRodon, (iii) phototrophy, predicted using the presence of bacteriochlorophyll synthesis genes, (iv) carbon fixation, predicted using the presence of key genes, (v) genome GC content and (vi) genome size computed directly from genomic data. See Methods for details on how these traits were inferred.

GC content and genome size were calculated directly from genomic data and included because these properties are associated with ecological attributes, from environmental tolerances to oxygen, light and temperature [49–52] to specific metabolisms [53] and ecological strategies [54, 55]. Both genome size and GC content were continuous values ranging from 0.22 to 25 Mbp and 15%–77%, respectively, across the 50 745 bacterial genomes. The taxa with very large genomes included many *Actinobacteriota, Myxococcota*, and specific clades of *Proteobacteria* and *Cyanobacteria*, and those with smaller genomes were found across many phyla—most *Firmicutes*, some *Proteobacteria*, and phyla like *Omnitrophota* and *Patescibacteria* where small genome sizes are a characteristic trait [55, 56]. The genomes with the highest GC content were common in *Actinobacteriota, Proteobacteria*, and sister clades, whereas fewer and more specific subgroups of *Firmicutes, Proteobacteria*, and *Bacteroidota* contained low-GC genomes (Fig. 2 and Supplementary Table 1).

Maximum potential growth rate was included because it is a key feature distinguishing variation in general life history strategies across bacteria [57, 58] with maximum potential growth rate approximated using minimum potential doubling time predictions from gRodon v2 [42]. gRodon’s maximum potential growth rate is a vertically transmissible trait i.e. invariant for any given taxon [59], unlike *in situ* growth rates (i.e. actual growth rates) which can be highly variable depending on specific environmental conditions. The estimated maximum potential growth rates ranged from 0.01 to 25 h, with lower values indicating faster potential growth and shorter generation times. Taxa with low estimated generation times (<1 h) were relatively uncommon but phylogenetically widespread across anaerobic *Firmicutes* and aerobic *Proteobacteria*. Long estimated generation times (slowest maximum potential growth) were prevalent in many groups, including specific *Proteobacteria*, many *Actinobacteriota*, and *Gemmatimonadota* (Fig. 2). Taxa that have been isolated in vitro almost always have shorter predicted generation times (faster maximum potential growth rates) than uncultivated taxa from the same phylum whose genomes were assembled from metagenomes (Supplementary Fig. 6), highlighting a bias for faster growth among taxa that are readily cultivable [26].

The potential for autotrophic metabolism, or the ability to fix CO_2_, was estimated using an approach that incorporates information on both the presence of specific genes and pathway completeness [23] for two of the four known bacterial autotrophy pathways (Calvin Cycle and hydroxypropionate bi-cycle) which are reasonably well-characterized [22, 60]. This trait is encoded as a binary variable (either inferred to be capable or incapable of autotrophic CO_2_ fixation) when a genome contained sufficient completeness of either pathway. Using this approach, 9.3% (4627) of the bacterial genomes were inferred to be capable of autotrophic metabolism via either of the two measured pathways. As expected, the inferred capacity for autotrophic CO_2_ fixation was most prevalent among *Cyanobacteria* and *Proteobacteria*, and otherwise found in small numbers of taxa in *Actinobacteriota* [61] and sister phyla of the *Firmicutes* supergroup [62–65] (Fig. 2).

We screened all genomes for key genes and pathways to infer chlorophototrophy, the capacity to capture light energy using chlorophyll. ~6% (3223) of bacterial genomes contained chlorophyll synthesis genes ubiquitous in a genomic dataset of chlorophototrophs [37]. Consistent with the literature [17, 37, 66–68], the inferred capacity for chlorophototrophy was largely (but not always) restricted to taxa within the phyla *Proteobacteria, Cyanobacteria, Chloroflexota*, and other groups which were also inferred to be autotrophic (Fig. 2).

Oxygen tolerance was predicted from gene presence using a random forest model trained on characterized taxa from BacDive [7]. Oxygen tolerance was encoded as the probability each genome is aerobic (zero to one) and was included because tolerance of oxygen is a key determinant of metabolic strategies [69] and the potential environments in which taxa can thrive. As expected, oxygen tolerance is not universally conserved at the phylum level (Fig. 2). Aerobes are particularly prevalent in *Proteobacteria, Actinobacteriota*, and at least some clades of most other phyla, whereas taxa within *Firmicutes A, Desulfobacterota*, and subgroups of some *Bacteroidota* were almost exclusively inferred to be anaerobes.

### Identification of taxa with shared traits using Haar-like wavelets

**Figure 3:**
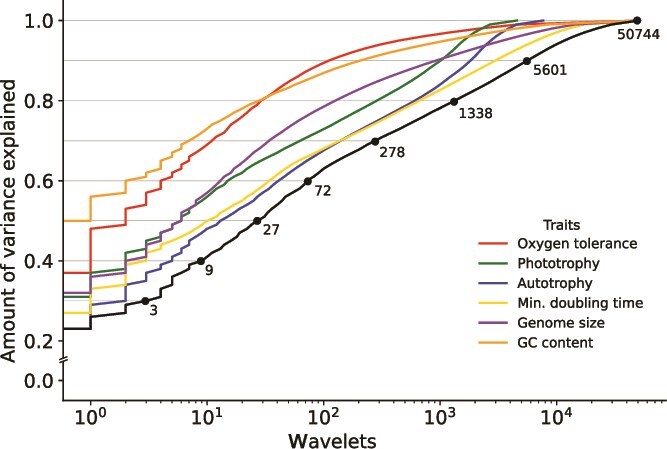
Per-coefficient variance explained for each trait versus the number of wavelets used to describe that variance. Each curve is constructed by sorting the wavelet coefficients for each trait by magnitude and summing the magnitudes. The intersection of horizontal bars at 10% variance intervals indicate the total number of unique wavelet coefficients (or phylogenetic splits) which explain that degree of total variance for the trait. The black line indicates the number of unique wavelets required to explain a particular degree of variance across all six traits; 100% of total trait variance is explained by n-1 wavelets (50 744) for the phylogenetic tree with n = 50 745 leaves. For the periodic table, we selected a threshold of 60% variance explained, which yielded a total of 1307 cells, or groupings of bacteria, with the 272 cells which contained the largest number of unique genomes (~80% out of the 50 745 genomes) shown in Fig. 5, our periodic table.

To identify lineages with conserved traits across all 50 745 bacterial genomes (from the genome-derived inferences described above), we summarized trait-phylogeny relationships using the Haar-like wavelet projection (HWP). The HWP performs a phylogenetically derived transformation of trait values associated with species, or leaves, into measurements of the trait variation uniquely attributed to each interior node of the phylogeny (see [32] for specific methods). The variation attributed to a node is reflected in the magnitude of a “wavelet coefficient” computed by comparing the trait values among leaves of the node’s left and right subtrees. The wavelet coefficients normalized to fraction of total variance (Supplementary Fig. 7) reveal that, for the six traits considered in this study, trait variance is explained by relatively few internal nodes. Additionally, despite the six traits assessed here being estimated independently and selected without any expectation of relatedness, at least 25% of the variation in oxygen tolerance, potential growth rate, GC content, and genome size are attributed to the same split (clade c000130) at the common ancestor of slow-growing high GC aerobes with large genomes (*Actinobacteriota*) and fast-growing low-GC anaerobes with small genomes (most *Firmicutes*). Fewer than 100 wavelets are required to explain 60% or more of total trait variance (Fig. 3); 26 of these wavelets are shared across two, three, or four traits, which is significantly more than would be expected using a Brownian motion model (Supplementary Fig. 8). These results imply that trait changes frequently occur at the same phylogenetic nodes, perhaps due to coordinated evolution of traits [18]. Additionally, many of the phylogenetic splits with large wavelet coefficients reside at deeper nodes, often at approximately the phylum level of resolution (Supplementary Fig. 9). Collectively the HWP reveals that these six traits are often correlated, significant sources of variation are found deep in the phylogeny, and small numbers of splits can describe trait distributions accurately. Together these results indicate that a PTBT can describe a majority of the trait variance across a broad diversity of bacteria, even when considering multiple traits simultaneously.

Association of traits with one another and phylogenetic structure is a desirable condition given that the utility of the PTBT is tied to its visual complexity and information fidelity. The deconvolution of phylogeny-trait relationships provided by the HWP illustrate that trait distributions and co-occurrence are not combinatorially complex, and that trait-taxon relationships can be simplified in a manner which preserves variation in a PTBT. Other studies have also determined that complex bacterial traits often exhibit a high degree of phylogenetic conservatism [18, 33], but our data reveal that these trends are not expressed equally among the traits examined here. The small number of highly explanatory wavelets (Fig. 3 and SF  2) indicate that autotrophy and maximum potential growth rate, and to lesser extents phototrophy and genome size, have relatively weaker phylogenetic signals (and therefore may be more difficult to summarize from phylogeny alone). The different degrees of conservation in genome size and GC content (Fig. 3), measured directly from genomes, indicate that variation in phylogenetic conservation is biological and not simply due to inference errors. Among our traits, various explanations may explain varying degrees of phylogenetic association, from a history of horizontal transmission in the Calvin Cycle [22], to selection or drift altering genome size based on the specific biotic and abiotic factors present [54, 70–72]. Such variations make visual properties which depict trait co-occurrence and depth of phylogenetic conservation important components of the PTBT’s visual design.

**Figure 4:**
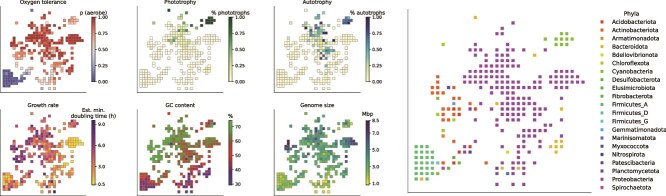
Data used to generate a layout of the periodic table diagram which combines trait and phylogenetic information using ENS-t-SNE. Each plot shows the distribution of six traits among the cells of the periodic table, with an additional plot to illustrate the phylum-level identities of the taxa within each of the cells. Each cell corresponds to a clade in the bacterial trait dataset (50 745 leaves total, 272 in this diagram) discovered by traversing the phylogeny until no sufficiently large Haar-like wavelets existed in any subtree. The median trait values from the resulting tips and a patristic distance matrix were provided to the ENS-t-SNE algorithm to generate a layout that included phylogenetic and trait information. The cells and their arrangement in these panels are identical to those shown in the final periodic table shown in Fig. 5, with this figure serving as a complement to Fig. 5 to help visualize how traits and taxonomic identities vary across the cells in the periodic table.

### Drafting a “periodic table” to visualize bacterial diversity in trait space

**Figure 5:**
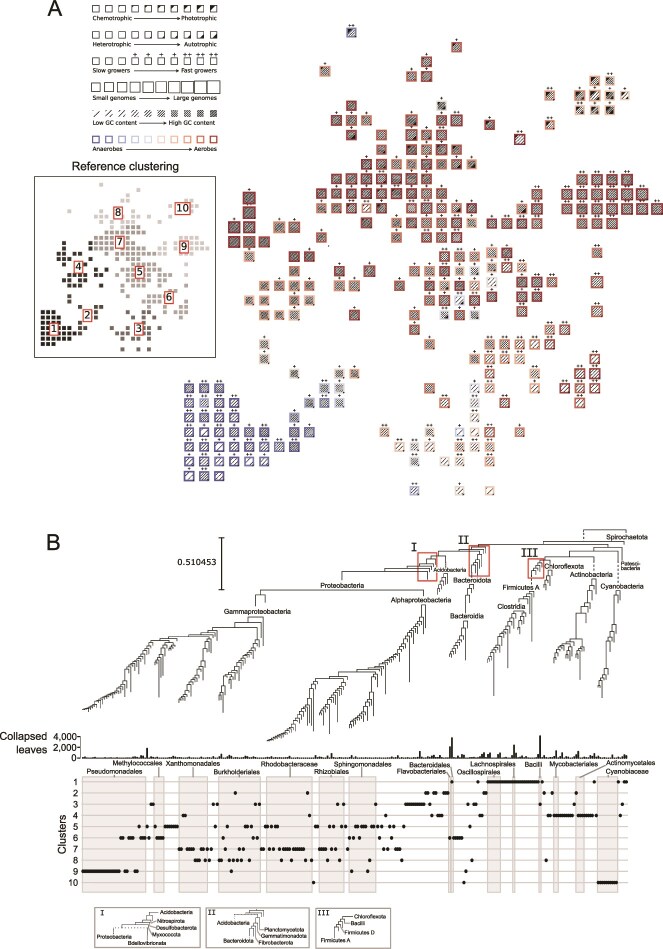
Periodic table of bacterial diversity. Functionally conserved taxonomic groups were identified by pruning the branches of a bacterial phylogeny with 50 745 leaves (species-level representative genomes) until an internal node associated with significant internal variance was discovered, essentially identifying the clades to put in a periodic table-like diagram. The layout of the table was generated by projecting a combination of trait and phylogenetic information into 2D space using ENS-t-SNE, then snapping points to a grid which preserved the nearby placement of clades with similar trait values and phylogenetic identity (Fig. 4). (A) A multi-trait illustration of the trait values for 272 groups of functionally homogeneous bacterial taxa discovered by the pruning algorithm (see Methods), each illustrated as a single cell. The periodic table is subdivided into 10 proximity-based clusters computed with k-means for the purposes of description, yielding a higher-level categorization of bacterial diversity in trait space. (B) The underlying pruned phylogeny of bacteria labelling the clades which appear in the diagram from panel A with some phylum and class-level annotations: relevant order-level annotations appear below the bar chart, which shows the number of leaves in the original phylogeny which were pruned, or collapsed, to create this phylogeny and the periodic table. The bottom panel shows the membership of each leaf (corresponding to cells in A) in reference clusters from A (inset). When necessary, branches are extended with dashed lines to avoid overlap of graphical elements. The three panels (I, II, and III) corresponding to the three red boxes label tightly spaced phyla.

The procedure for constructing the PTBT involves analyzing the wavelet coefficients to find parents of sister clades whose leaves *are not similar* with respect to each trait and therefore do not represent the functionally uniform (or nearly uniform) clades that we want the cells of the PTBT to represent. Our PTBT tries to maximize visual analogy to the original periodic table in addition to explaining most of the variance in the trait data: however, the amount of trait variance explained (TVE) is ultimately determined by a user’s desire to balance the number of summarized trait-taxonomy groups with TVE. In our example, a value near the inflection point of the wavelet magnitude versus variance explained distribution, 60%, (Fig. 3) maximizes TVE and keeps the number of cells in the PTBT manageable. After applying the HWP, the 72 unique nodes required to explain 60% of the trait variance were used to prune branches and create a simplified phylogeny containing junctions which contribute to TVE (Supplementary Fig. 10A and B). This simplified phylogeny is the backbone of our example PTBT: its “leaves” are quantitatively discovered clades of bacteria, represented in the tree by their most recent common ancestor, which have minimal descendent trait variation (Supplementary Fig. 10C). Although the HWP-based method of discovering these groups is our core finding, we generate an actual “periodic table”-like diagram of bacterial taxa with shared traits to illustrate how visualization can facilitate interpretation (Supplementary Fig. 10D). Generating this diagram first involved placing each clade into a two dimensional visualization. Techniques like t-SNE [73] can map high-dimensional points into two-dimensional space; here, t-SNE allows us to emulate the groupings used in the periodic table of elements by using each clade’s median trait values to locate it near functionally similar clades. To address the additional challenge of incorporating relational phylogenetic information to the layout, we used ENS-t-SNE [45], an extension of t-SNE which can generate a joint mapping of both pairwise phylogenetic distances and trait data into a single space. The resulting layout combines phylogenetic information with trait information to gather cells which are similar based on phylogenetic and/or trait-based distance (Fig. 4). Finally, a PTBT is drawn by summarizing the traits of each functionally uniform clade into a cell using this layout (Fig. 5A), and providing the tree representing the phylogenetic relationships among the cells (Fig. 5B). The median trait values for each clade are drawn with different graphical elements of the cells in Fig. 5A, our initial design of an empirically derived PTBT, including a diagram of the phylogeny which resulted from HWP-based collapsing to illustrate the taxonomic groups which constitute the cells in the table (Fig. 5B).

The PTBT illustrates the distributions of traits across bacterial phylogenetic diversity and facilitates the interpretation of trait co-occurrence, conservation, and divergence patterns in specific taxa. First, the PTBT illustrates the variation in the phylogenetic depths at which trait combinations are conserved. The PTBT assigns *Proteobacteria, Firmicutes A*, and *Actinobacteriota* more cells (172, 24, and 21 respectively) because traits vary more in these phyla versus *Myxococcota, Cyanobacteria, Spirochaetota, Patescibacteria*, and other phyla with less variation in the traits assessed here (Fig. 4). Second, the layout identifies when a particular set of traits has emerged in multiple phylogenetically distinct taxa, e.g. distinguishing primarily oxygen-tolerant *Actinobacteriota* with high GC contents and large genomes (cluster 4, Fig. 5A) from phylogenetically distinct but otherwise similar pseudomonads (clusters 6 and 9, Fig. 5A). The PTBT also illustrates where related taxa have distinct traits: two examples are the oxygen-tolerating photoautotrophic *Cyanobacteriota* (cluster 10, Fig. 5A) and chemoheterotrophic *Bacteroidota* (cluster 6, Fig. 5A), each of which are far from their sister clades of chemoheterotrophic low-GC anaerobes (*Bacteroidales* in *Bacteroidota*; *Vampirovibrionia* in *Cyanobacteria*, both in cluster 1 of Fig. 5A). Finally, the PTBT makes it possible to identify the taxonomic underpinnings of high-level associations between traits. For example, the visual separation of small, blue, lightly hashed cells (cluster 1 and 2; low-GC/small-genome anaerobes) and large, red, densely hashed cells (regions 4, 5, 7, 8, and 9; high-GC/large-genome aerobes) is driven by the distinction between *Firmicutes* and *Actinobacteria/Proteobacteria*, a result consistent with previous work [70]. Essentially, the PTBT maps trait distributions into visual components of the cells and layout; different distances, sizes, colors, and other visual elements of the cells enable users to find bacteria with particular groups of traits, identify phylogenetic and functional distinctions between taxonomic groups, and assess the relative diversity of particular groups in trait space by examining both the number of cells in a group and their placement relative to one another.

## Conclusions

Our PTBT uses a phylogenetic approach i.e. quantitative, efficient, interpretable, and flexible enough to be applied to various collections of traits and phylogenetic trees, continuously integrate trait data with growing phylogenetic databases, and provide data-driven insights into trait distributions at large scales. Developing trait-based approaches with these properties is explicitly identified as a key step in the development of microbial trait frameworks [74]; in our work, many of these desirable properties come from the HWP. Although the HWP is not the only means of quantitatively defining trait-taxonomy distributions [11, 18, 75, 76], it is better suited for the task of algorithmically identifying functionally uniform groups than individual statistics for measuring trait-phylogeny relationships like Pagel’s lambda [77], Blomberg’s K [78] or even consenTRAIT [18] because it decomposes trait variance to specific nodes rather than globally measuring trait-phylogeny relationships. Phylogenetic approaches like phylogenetic generalized least squares [79] or ancestral state reconstruction [80] can infer per-node trait states, but these states are inferential rather than descriptive and therefore embed a particular evolutionary model to assess how an evolutionary model explains the evolutionary history of a trait. The assessment of evolutionary models for particular bacterial traits, although critical, is not the goal of the PTBT, which we conceive as an efficient, flexible, and descriptive approach to describe how trait values are distributed relative to a given phylogenetic structure. The dependence of our PTBT and the HWP on a fixed phylogeny without uncertainties is a caveat worth acknowledging [32, 81], but there is no reason this approach could not be applied to bootstraps or posterior distributions for a particular tree to incorporate phylogenetic uncertainty into future analyses.

Our use of phylogenetic and empirical genome-based information to assess trait distributions in the PTBT is a deliberate attempt to provide data-driven (rather than conceptual or anecdotal) evidence for the presence of associated bacterial traits, often termed “ecological strategies.” The YAS framework [9, 10, 82], which adapts Grime’s CSR theory [83] to microorganisms, is among the most widely known conceptual frameworks for the ecological strategies of microbes. Both the CSR and YAS frameworks can be characterized as descriptions of how fitness is distributed across trait space: although many theoretical trait combinations are possible, CSR and YAS hypothesize that environments, evolutionary history, and physiological constraints limit which combinations of traits confer high fitness. Our thesis for the PTBT, that a joint phylogenetic-trait space can be effectively represented with a small amount of data, is based on the same idea. In both views, trait space is not evenly occupied but structured by peaks of fitness (and therefore higher frequencies of organisms) where viable strategies recur. Our quantitative PTBT realizes this idea by identifying clusters of co-occurring traits in multidimensional trait space which could define distinct ecological strategies while simultaneously quantifying the distribution of these ecological strategies and associated traits among bacterial taxa. By constructing a PTBT using environmental factors known to be associated with compositional shifts, or hypothesized to be involved a particular strategy, our work’s empirical classification of taxa into trait-based groups could provide a quantitative starting point for assessing how biogeographic patterns in bacterial taxa are explained by their trait distributions.

Our PTBT method applies genome-based trait inference methods, the HWP, and ENS-t-SNE to estimate traits across bacteria and then identify and organize groups of bacteria into a visual framework that captures trait co-occurrence, phylogenetic conservation, and phylogenetic similarity. The PTBT could be used to further characterize complex but important traits, including those from CSR or YAS theory like stress tolerance and resource acquisition, because our methods are flexible. In our example, which by no means a canonical periodic table, we used autotrophy, phototrophy, oxygen tolerance, growth rate, and genomic descriptors and found that we can effectively organize large swaths of bacterial diversity in trait space with the PTBT to create an informative visual summarization of selected bacterial taxon-trait relationships. We expect that our approach could generate meaningful simplifications for other trait-taxon associations, particularly as new traits become possible to estimate from genomic data. In particular, we think that the HWP could help propose new representations of trait-phylogeny relationships in microbial datasets. With adaptation, these methods could also help integrate trait data with genome databases like GTDB [6] to unify microbial functional, phylogenetic, and genomic information in a single interface.

Although we believe our efforts to synthesize traits, phylogenetic information, and visualization techniques have many realized and unrealized benefits, we acknowledge that our workflow is inherently reductive and could be improved. We have only visualized six traits with a specified degree of variance and largely ignored potential errors in both trait estimation methods and the phylogeny itself. In addition to improvements to trait estimations and phylogenetic inferences, whether the PTBT provides verifiable explanations of the realized patterns in ecosystems should be critically assessed in further work. In the meantime, we hope the PTBT will inspire development of new tools to summarize, assess, and validate our current knowledge—and assumptions about—the taxon-trait associations upon which many analyses in microbial ecology explicitly or implicitly rely. Efficiently finding and presenting evidence for a particular trait’s presence in taxa, the essential task the PTBT is designed to do, serves an important role in microbial ecology, and our work provides an initial demonstration of how such quantitative trait organization schemes can be designed and could inform evidence-based exploration of complex trait distributions across phylogenetically diverse and poorly characterized microbial communities.

## Supplementary Material

wraf289_Supplemental_Files

## Data Availability

Intermediate data files and code used to generate figures and analyses are available on GitHub: https://github.com/realmichaelhoffert/bacterial_periodic_table/tree/main. The oxygen tolerance model is available at on figshare (https://dx.doi.org/10.6084/m9.figshare.30610709).

## References

[1] Schwerdtfeger P, Smits OR, Pyykkö P. The periodic table and the physics that drives it. *Nat Rev Chem* 2020;4:359–80. 10.1038/s41570-020-0195-y 37127952

[2] Gänzle M . The periodic table of fermented foods: limitations and opportunities. *Appl Microbiol Biotechnol* 2022;106:2815–26. 10.1007/s00253-022-11909-y 35412130

[3] P JB . A nuclear chocolate box: the periodic table of nuclear medicine. *Dalton Trans* 2015;44:4819–44. 10.1039/C4DT02846E 25406520 PMC6205633

[4] Lengler R, Eppler MJ. Towards a periodic table of visualization methods of management. In: Alam M (ed.), Proc. IASTED Int. Conf. Graph. Vis. Eng*.* 2007. USA: ACTA Press, 2007, pp. 83–8.

[5] Xia B, Yanai I. A periodic table of cell types. *Development* 2019;146:dev169854. 10.1242/dev.169854 31249003 PMC6602355

[6] Parks DH, Chuvochina M, Rinke C. et al. GTDB: an ongoing census of bacterial and archaeal diversity through a phylogenetically consistent, rank normalized and complete genome-based taxonomy. *Nucleic Acids Res* 2022;50:D785–94. 10.1093/nar/gkab776 34520557 PMC8728215

[7] Reimer LC, Vetcininova A, Carbasse JS. et al. BacDive in 2019: bacterial phenotypic data for high-throughput biodiversity analysis. *Nucleic Acids Res* 2019;47:D631–6. 10.1093/nar/gky879 30256983 PMC6323973

[8] Louca S, Polz MF, Mazel F. et al. Function and functional redundancy in microbial systems. *Nat Ecol Evol* 2018;2:936–43. 10.1038/s41559-018-0519-1 29662222

[9] Wood JL, Malik AA, Greening C. et al. Rethinking CSR theory to incorporate microbial metabolic diversity and foraging traits. *ISME J* 2023;17:1793–7. 10.1038/s41396-023-01486-x 37596410 PMC10579239

[10] Rodriguez LC, Murúa JM, Marschmann GL. et al. Y-A+S Is the New Y-A-S: Updating Microbial Life History Tradeoffs with Comparative Genomics. preprint, *Research Square* 2025;**2 May 2025.** 10.21203/rs.3.rs-6486618/v1

[11] Karaoz U, Brodie EL. microTrait: a toolset for a trait-based representation of microbial genomes. *Front Bioinforma* 2022;2:918853. 10.3389/fbinf.2022.918853 PMC9580909 36304272

[12] Winemiller KO, Fitzgerald DB, Bower LM. et al. Functional traits, convergent evolution, and periodic tables of niches. *Ecol Lett* 2015;18:737–51. 10.1111/ele.12462 26096695 PMC4744997

[13] Garrity GM . A new genomics-driven taxonomy of bacteria and archaea: are we there yet? *J Clin Microbiol* 2016;54:1956–63. 10.1128/JCM.00200-16 27194687 PMC4963521

[14] Godfray HCJ . Challenges for taxonomy. *Nature* 2002;417:17–9. 10.1038/417017a 11986643

[15] Thomas CM, Nielsen KM. Mechanisms of, and barriers to, horizontal gene transfer between bacteria. *Nat Rev Microbiol* 2005;3:711–21. 10.1038/nrmicro1234 16138099

[16] Parks DH, Chuvochina M, Waite DW. et al. A standardized bacterial taxonomy based on genome phylogeny substantially revises the tree of life. *Nat Biotechnol* 2018;36:996–1004. 10.1038/nbt.4229 30148503

[17] Sánchez-Baracaldo P, Cardona T. On the origin of oxygenic photosynthesis and *cyanobacteria*. *New Phytol* 2020;225:1440–6. 10.1111/nph.16249 31598981

[18] Martiny AC, Treseder K, Pusch G. Phylogenetic conservatism of functional traits in microorganisms. *ISME J* 2013;7:830–8. 10.1038/ismej.2012.160 23235290 PMC3603392

[19] Ramoneda J, Hoffert M, Stallard-Olivera E. et al. Leveraging genomic information to predict environmental preferences of bacteria. *ISME J* 2024;18:wrae195. 10.1093/ismejo/wrae195 39361898 PMC11488383

[20] Barberán A, Velazquez HC, Jones S. et al. Hiding in plain sight: mining bacterial species records for phenotypic trait information. *mSphere* 2017;2:e00237–17. 10.1128/mSphere.00237-17 28776041 PMC5541158

[21] Madin JS, Nielsen DA, Brbic M. et al. A synthesis of bacterial and archaeal phenotypic trait data. *Sci Data* 2020;7:170. 10.1038/s41597-020-0497-4 32503990 PMC7275036

[22] Asplund-Samuelsson J, Hudson EP. Wide range of metabolic adaptations to the acquisition of the Calvin cycle revealed by comparison of microbial genomes. *PLoS Comput Biol* 2021;17:e1008742. 10.1371/journal.pcbi.1008742 33556078 PMC7895386

[23] Garritano AN, Song W, Thomas T. Carbon fixation pathways across the bacterial and archaeal tree of life. *PNAS Nexus* 2022;1:pgac226. 10.1093/pnasnexus/pgac226 36712370 PMC9802188

[24] Dos Santos PC, Fang Z, Mason SW. et al. Distribution of nitrogen fixation and nitrogenase-like sequences amongst microbial genomes. *BMC Genomics* 2012;13:162. 10.1186/1471-2164-13-162 22554235 PMC3464626

[25] Su Z, Wen D. Characterization of antibiotic resistance across Earth’s microbial genomes. *Sci Total Environ* 2022;816:151613. 10.1016/j.scitotenv.2021.151613 34774941

[26] Weissman JL, Hou S, Fuhrman JA. Estimating maximal microbial growth rates from cultures, metagenomes, and single cells via codon usage patterns. *Proc Natl Acad Sci* 2021;118:e2016810118. 10.1073/pnas.2016810118 33723043 PMC8000110

[27] Cimen E, Jensen SE, Buckler ES. Building a tRNA thermometer to estimate microbial adaptation to temperature. *Nucleic Acids Res* 2020;48:12004–15. 10.1093/nar/gkaa1030 33196821 PMC7708079

[28] Li G, Rabe KS, Nielsen J. et al. Machine learning applied to predicting microorganism growth temperatures and enzyme catalytic optima. *ACS Synth Biol* 2019;8:1411–20. 10.1021/acssynbio.9b00099 31117361

[29] Sauer DB, Wang D-N. Predicting the optimal growth temperatures of prokaryotes using only genome derived features. *Bioinformatics* 2019;35:3224–31. 10.1093/bioinformatics/btz059 30689741 PMC6748728

[30] Ramoneda J, Stallard-Olivera E, Hoffert M. et al. Building a genome-based understanding of bacterial pH preferences. *Sci Adv* 2023;9:eadf8998. 10.1126/sciadv.adf8998 37115929 PMC10146879

[31] Jabłońska J, Tawfik DS. The number and type of oxygen-utilizing enzymes indicates aerobic vs. anaerobic phenotype. *Free Radic Biol Med* 2019;140:84–92. 10.1016/j.freeradbiomed.2019.03.031 30935870

[32] Gorman ED, Lladser ME. Interpretable metric learning in comparative metagenomics: the adaptive Haar-like distance. *PLoS Comput Biol* 2024;20:e1011543. 10.1371/journal.pcbi.1011543 38768195 PMC11142682

[33] Goberna M, Verdú M. Predicting microbial traits with phylogenies. *ISME J* 2016;10:959–67. 10.1038/ismej.2015.171 26371406 PMC4796935

[34] Parks DH, Imelfort M, Skennerton CT. et al. CheckM: assessing the quality of microbial genomes recovered from isolates, single cells, and metagenomes. *Genome Res* 2015;25:1043–55. 10.1101/gr.186072.114 25977477 PMC4484387

[35] Hyatt D, Chen G, LoCascio PF. et al. Prodigal: prokaryotic gene recognition and translation initiation site identification. *BMC Bioinformatics* 2010;11:119. 10.1186/1471-2105-11-119 20211023 PMC2848648

[36] Kramer O . Scikit-learn. In: Kramer O. (ed.), Machine Learning for Evolution Strategies. Cham: Springer International Publishing, 2016, 45–53.

[37] Thiel V, Tank M, Bryant DA. Diversity of chlorophototrophic bacteria revealed in the omics era. *Annu Rev Plant Biol* 2018;69:21–49. 10.1146/annurev-arplant-042817-040500 29505738

[38] Zimmermann J, Kaleta C, Waschina S. Gapseq: informed prediction of bacterial metabolic pathways and reconstruction of accurate metabolic models. *Genome Biol* 2021;22:81. 10.1186/s13059-021-02295-1 33691770 PMC7949252

[39] Caspi R, Billington R, Keseler IM. et al. The MetaCyc database of metabolic pathways and enzymes - a 2019 update. *Nucleic Acids Res* 2020;48:D445–53. 10.1093/nar/gkz862 31586394 PMC6943030

[40] Kanehisa M, Goto S. KEGG: Kyoto Encyclopedia of genes and genomes. *Nucleic Acids Res* 2000;28:27–30. 10.1093/nar/28.1.27 10592173 PMC102409

[41] Momper L, Jungbluth SP, Lee MD. et al. Energy and carbon metabolisms in a deep terrestrial subsurface fluid microbial community. *ISME J* 2017;11:2319–33. 10.1038/ismej.2017.94 28644444 PMC5607374

[42] Weissman JL, Peras M, Barnum TP. et al. Benchmarking community-wide estimates of growth potential from metagenomes using codon usage statistics. *mSystems* 2022;7:e0074522–2. 10.1128/msystems.00745-22 36190138 PMC9600850

[43] Camacho C, Coulouris G, Avagyan V. et al. BLAST+: architecture and applications. *BMC Bioinformatics* 2009;10:421. 10.1186/1471-2105-10-421 20003500 PMC2803857

[44] Vieira-Silva S, Rocha EPC. The systemic imprint of growth and its uses in ecological (meta)genomics. *PLoS Genet* 2010;6:e1000808. 10.1371/journal.pgen.1000808 20090831 PMC2797632

[45] Miller J, Huroyan V, Navarrete R. et al. ENS-t-SNE: Embedding neighborhoods simultaneously using t-SNE. In: Fujishiro I (ed.), 2024 IEEE 17th Pac. Vis. Conf. PacificVis. Tokyo: IEEE, 2024, pp. 222–31. 10.1109%2FPacificVis60374.2024.00032.

[46] Waskom ML . Seaborn: statistical data visualization. *J Open Source Softw* 2021;6:3021. 10.21105/joss.03021

[47] Harris CR, Millman KJ, van der Walt SJ. et al. Array programming with NumPy. *Nature* 2020;585:357–62. 10.1038/s41586-020-2649-2 32939066 PMC7759461

[48] Hunter JD . Matplotlib: a 2D graphics environment. *Comput Sci Eng* 2007;9:90–5. 10.1109/MCSE.2007.55

[49] Aslam S, Lan X, Zhang B. et al. Aerobic prokaryotes do not have higher GC contents than anaerobic prokaryotes, but obligate aerobic prokaryotes have. *BMC Evol Biol* 2019;19:35. 10.1186/s12862-019-1365-8 30691392 PMC6350292

[50] Hildebrand F, Meyer A, Eyre-Walker A. Evidence of selection upon genomic GC-content in bacteria. *PLoS Genet* 2010;6:e1001107. 10.1371/journal.pgen.1001107 20838593 PMC2936529

[51] Hurst LD, Merchant AR. High guanine-cytosine content is not an adaptation to high temperature: a comparative analysis amongst prokaryotes. *Proc R Soc B Biol Sci* 2001;268:493–7. 10.1098/rspb.2000.1397 PMC1088632 11296861

[52] Sabath N, Ferrada E, Barve A. et al. Growth temperature and genome size in bacteria are negatively correlated, suggesting genomic streamlining during thermal adaptation. *Genome Biol Evol* 2013;5:966–77. 10.1093/gbe/evt050 23563968 PMC3673621

[53] Mcewan CEA, Gatherer D, Mcewan NR. Nitrogen-fixing aerobic bacteria have higher genomic GC content than non-fixing species within the same genus. *Hereditas* 1998;128:173–8. 10.1111/j.1601-5223.1998.00173.x 9687237

[54] Brewer TE, Handley KM, Carini P. et al. Genome reduction in an abundant and ubiquitous soil bacterium ‘*Candidatus Udaeobacter copiosus*’. *Nat Microbiol* 2016;2:1–7. 10.1038/nmicrobiol.2016.198 27798560

[55] Tian R, Ning D, He Z. et al. Small and mighty: adaptation of superphylum *Patescibacteria* to groundwater environment drives their genome simplicity. *Microbiome* 2020;8:51. 10.1186/s40168-020-00825-w 32252814 PMC7137472

[56] Williams TJ, Allen MA, Berengut JF. et al. Shedding light on microbial “dark matter”: insights into novel *Cloacimonadota* and *Omnitrophota* from an Antarctic lake. *Front Microbiol* 2021;12:741077. 10.3389/fmicb.2021.741077 34707591 PMC8542988

[57] Belliveau NM, Chure G, Hueschen CL. et al. Fundamental limits on the rate of bacterial growth and their influence on proteomic composition. *Cell Syst* 2021;12:924–944.e2. 10.1016/j.cels.2021.06.002 34214468 PMC8460600

[58] Dragone NB, Hoffert M, Strickland MS. et al. Taxonomic and genomic attributes of oligotrophic soil bacteria. *ISME Commun* 2024;4:ycae081. 10.1093/ismeco/ycae081 38988701 PMC11234899

[59] Xu L, Zakem E, Weissman JL. Improved maximum growth rate prediction from microbial genomes by integrating phylogenetic information. *Nat Commun* 2025;16:4256. 10.1038/s41467-025-59558-9 40335538 PMC12059116

[60] Berg IA . Ecological aspects of the distribution of different autotrophic CO_2_ fixation pathways. *Appl Environ Microbiol* 2011;77:1925–36. 10.1128/AEM.02473-10 21216907 PMC3067309

[61] Lee JH, Park DO, Park SW. et al. Expression and regulation of ribulose 1,5-bisphosphate carboxylase/oxygenase genes in *mycobacterium* sp. strain JC1 DSM 3803. *J Microbiol* 2009;47:297–307. 10.1007/s12275-008-0210-3 19557347

[62] Caldwell PE, MacLean MR, Norris PR. Ribulose bisphosphate carboxylase activity and a Calvin cycle gene cluster in *Sulfobacillus* species. *Microbiology* 2007;153:2231–40. 10.1099/mic.0.2007/006262-0 17600067

[63] Zakharchuk LM, Egorova MA, Tsaplina IA. et al. Activity of the enzymes of carbon metabolism in *Sulfobacillus sibiricus* under various conditions of cultivation. *Microbiology* 2003;72:553–7. 10.1023/A:1026039132408 14679899

[64] Berg IA, Keppen OI, Krasil'nikova EN. et al. Carbon metabolism of filamentous anoxygenic phototrophic bacteria of the family *Oscillochloridaceae*. *Microbiology* 2005;74:258–64. 10.1007/s11021-005-0060-5 16119842

[65] Narsing Rao MP, Luo Z, Dong Z. et al. Metagenomic analysis further extends the role of *Chloroflexi* in fundamental biogeochemical cycles. *Environ Res* 2022;209:112888. 10.1016/j.envres.2022.112888 35143804

[66] Shih PM, Ward LM, Fischer WW. Evolution of the 3-hydroxypropionate bicycle and recent transfer of anoxygenic photosynthesis into the *Chloroflexi*. *Proc Natl Acad Sci* 2017;114:10749–54. 10.1073/pnas.1710798114 28923961 PMC5635909

[67] Ward LM, Shih PM. Phototrophy and carbon fixation in *Chlorobi* postdate the rise of oxygen. *PLoS One* 2022;17:e0270187. 10.1371/journal.pone.0270187 35913911 PMC9342728

[68] Fischer WW, Hemp J, Johnson JE. Evolution of oxygenic photosynthesis. *Annu Rev Earth Planet Sci* 2016;44:647–83. 10.1146/annurev-earth-060313-054810

[69] Khademian M, Imlay JA. How microbes evolved to tolerate oxygen. *Trends Microbiol* 2021;29:428–40. 10.1016/j.tim.2020.10.001 33109411 PMC8043972

[70] Nielsen DA, Fierer N, Geoghegan JL. et al. Aerobic bacteria and archaea tend to have larger and more versatile genomes. *Oikos* 2021;130:501–11. 10.1111/oik.07912

[71] Nishida H . Evolution of genome base composition and genome size in bacteria. *Front Microbiol* 2012;3:420. 10.3389/fmicb.2012.00420 23230432 PMC3515811

[72] Rodríguez-Gijón A, Nuy JK, Mehrshad M. et al. A genomic perspective across earth’s microbiomes reveals that genome size in archaea and bacteria is linked to ecosystem type and trophic strategy. *Front Microbiol* 2022;12:761869. 10.3389/fmicb.2021.761869 35069467 PMC8767057

[73] van der ML, Hinton G. Visualizing data using t-SNE. *J Mach Learn Res* 2008;9:2579–605.

[74] Lajoie G, Kembel SW. Making the most of trait-based approaches for microbial ecology. *Trends Microbiol* 2019;27:814–23. 10.1016/j.tim.2019.06.003 31296406

[75] Royalty TM, Steen AD. Quantitatively partitioning microbial genomic traits among taxonomic ranks across the microbial tree of life. *mSphere* 2019;4:10.1128/msphere.00446-19. 10.1128/msphere.00446-19 PMC6714891 31462411

[76] Shan X, Goyal A, Gregor R. et al. Annotation-free discovery of functional groups in microbial communities. *Nat Ecol Evol* 2023;7:716–24. 10.1038/s41559-023-02021-z 36997739

[77] Pagel M . Inferring the historical patterns of biological evolution. *Nature* 1999;401:877–84. 10.1038/44766 10553904

[78] Blomberg SP, Garland T, Ives AR. Testing for phylogenetic signal in comparative data: behavioral traits are more labile. *Evol Int J Org Evol* 2003;57:717–45. 10.1111/j.0014-3820.2003.tb00285.x 12778543

[79] Martins EP, Hansen TF. Phylogenies and the comparative method: a general approach to incorporating phylogenetic information into the analysis of interspecific data. *Am Nat* 1997;149:646–67. 10.1086/286013

[80] Pagel M . The maximum likelihood approach to reconstructing ancestral character states of discrete characters on phylogenies. *Syst Biol* 1999;48:612–22. 10.1080/106351599260184

[81] Gorman E, Lladser ME. Sparsification of large ultrametric matrices: insights into the microbial tree of life. *Proc R Soc Math Phys Eng Sci* 2023;479:20220847. 10.1098/rspa.2022.0847

[82] Malik AA, Martiny JBH, Brodie EL. et al. Defining trait-based microbial strategies with consequences for soil carbon cycling under climate change. *ISME J* 2020;14:1–9. 10.1038/s41396-019-0510-0 31554911 PMC6908601

[83] Grime JP . Evidence for the existence of three primary strategies in plants and its relevance to ecological and evolutionary theory. *Am Nat* 1977;111:1169–94. 10.1086/283244

