# Supplementary material for: Constructing a “periodic table” of bacteria to map diversity in trait space
Source: ISME J. 2026 Jan 1;20(1):wraf289. doi: 10.1093/ismejo/wraf289 (PMC12815261; doi:10.1093/ismejo/wraf289)
Supplement: wraf289_Supplemental_Files [file wraf289_supplemental_files.zip › 2025_SupplementaryInformation_wraf289.pdf]

Supplementary Table 1
Tab-delimited file containing rows describing trait values for 50,745 representative genomes from GTDB r207. The columns are: 1) genome accession 2) oxygen tolerance 3) phototrophy 4) autotrophy 5) growth rate 6) genome size 7) GC percentage. Data are described in detail in Materials and Methods.

Supplementary Figures 1-10
Figures containing additional detail pertaining to datasets, analyses, models, and distributions of traits.

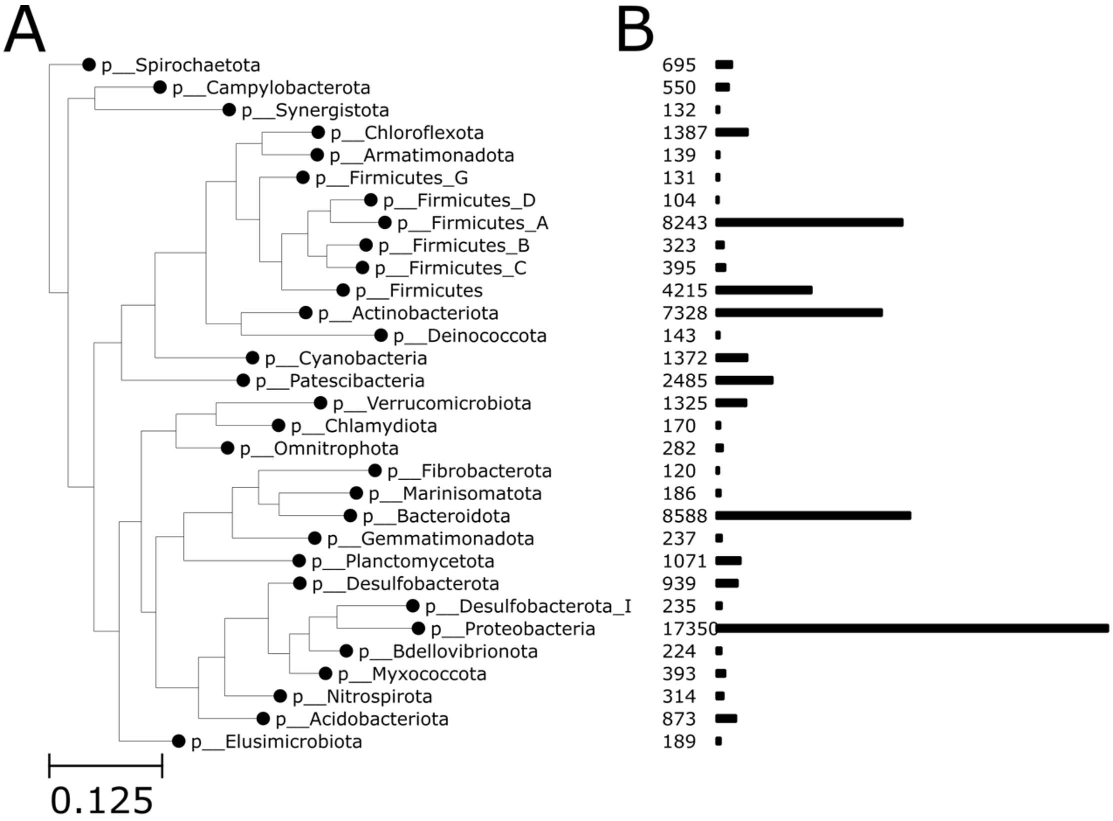

SF1 – (A) Reference GTDB phylogeny of 31 bacterial phyla with at least 100 genomes used in this study. (B) Bars and values showing the number of representative, ‘species’-level GTDB genomes per phylum.

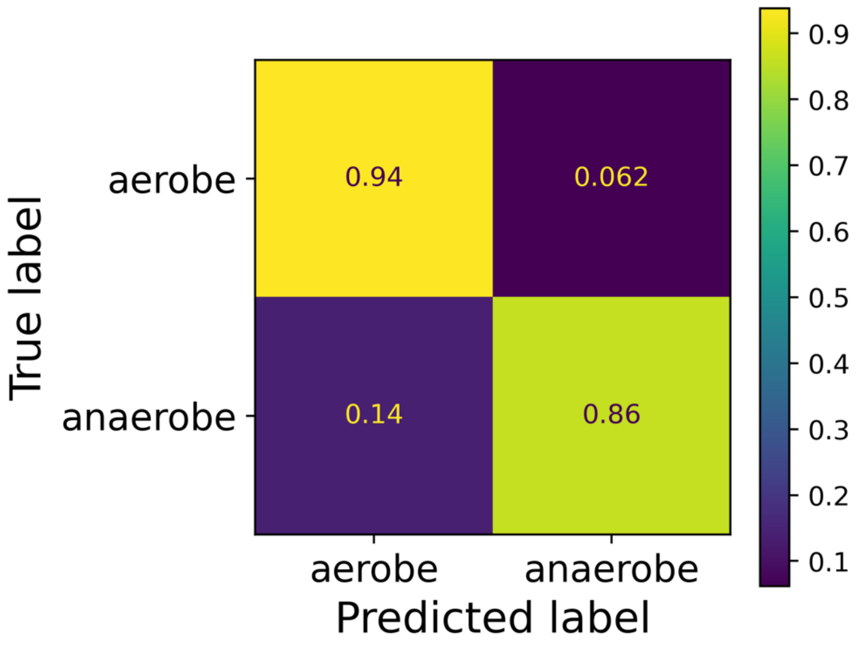

SF2 – Confusion matrix for the random forest model used to infer oxygen tolerance evaluated on the withheld test dataset (n=664). The matrix shows the fraction of true and false predictions for true members of each class, indicating the total percent of correct (upper-left, bottom-right) or incorrect (bottom-left, upper-right) classifications across categories.

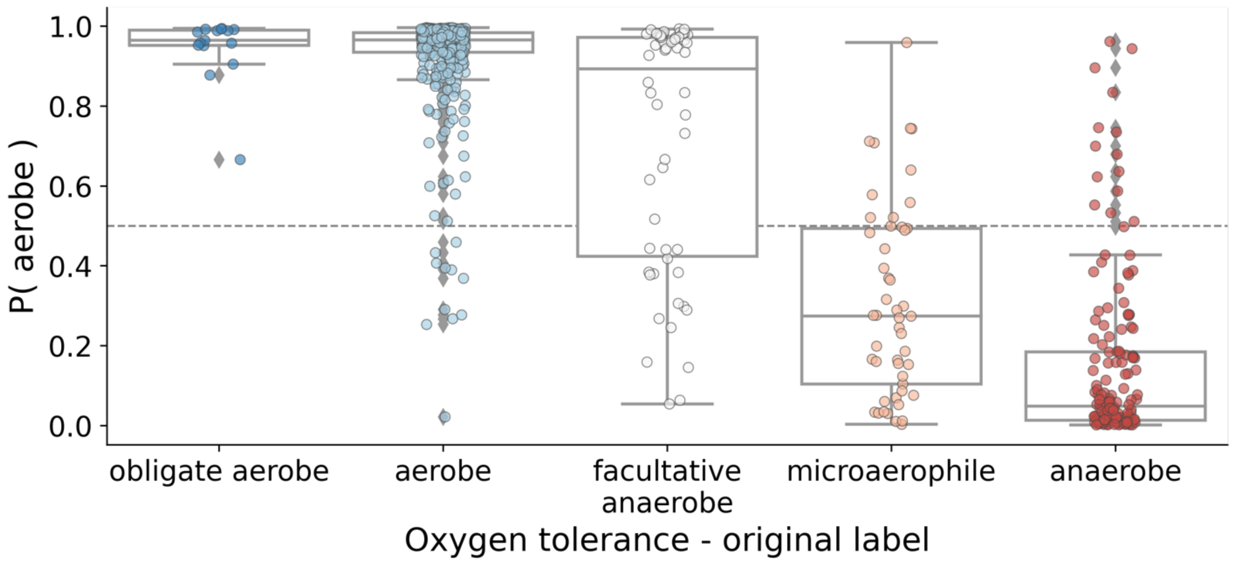

SF3 – Comparison of the random forest's prediction probability versus the original BacDive oxygen tolerance category for 664 bacteria in the test dataset. The y-axis shows the predicted probability of being oxygen tolerant, or probability of classification as "aerobe," produced by the model. The x-axis labels each bacterial taxon into the original BacDive categories, which were

grouped into “aerobe” or “anaerobe” based on the class they were most similar to. This plot illustrates that the model can successfully distinguish intermediate degrees of oxygen tolerance.

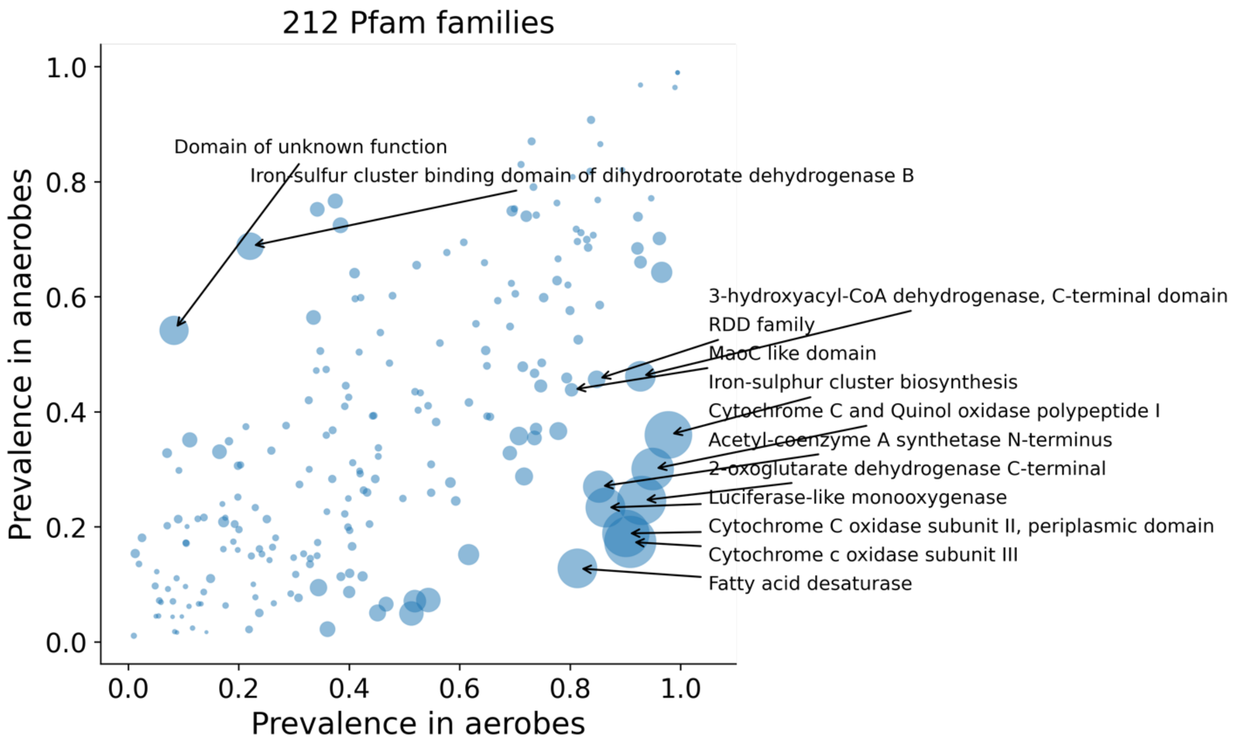

SF4– Difference in prevalence of 212 Pfam families used by our random forest to predict oxygen tolerance in aerobic bacteria (x-axis) and anaerobic bacteria (y-axis) from the training dataset. Points are sized by random forest feature importance to illustrate that points with larger differences in prevalence are more important to distinguishing aerobes from anaerobes. High-importance genes are annotated with descriptions from the Pfam database.

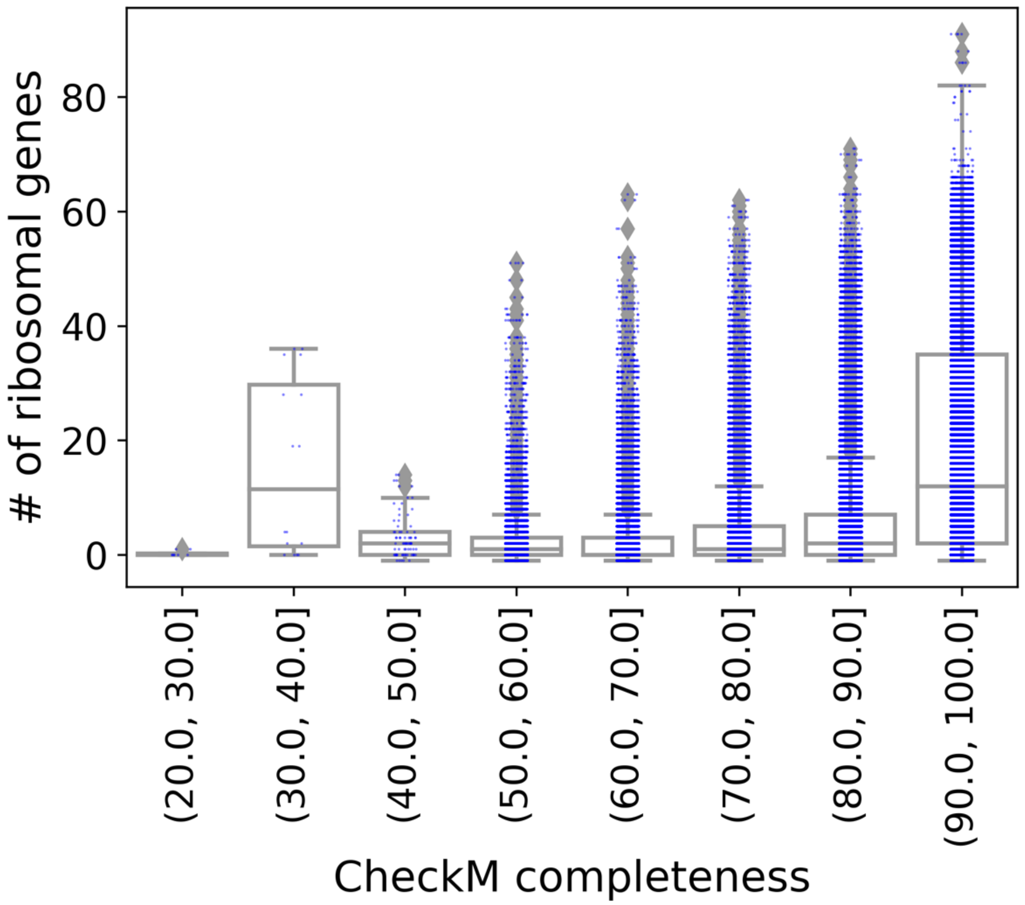

SF5 - Number of ribosomal proteins versus genome completeness for all v207 GTDB representative genomes showing that the number of ribosomal proteins detected decreases as genome completeness is reduced. Genome completeness likely influences the accuracy of minimum doubling time estimates produced by gRodon.

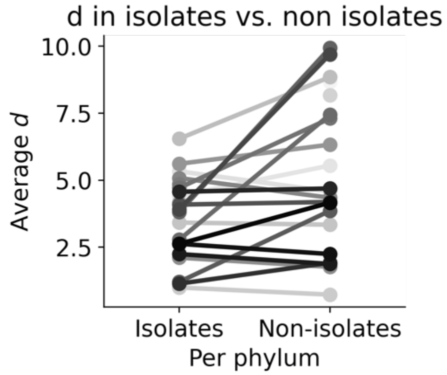

SF6– Average predicted minimal doubling times (*d*) in hours from gRodon v2 in GTDB genomes, divided between genomes labeled as “environmental” (non-isolates, or metagenome-assembled genomes), or “other” (isolates) for the 31 largest bacterial phyla shows substantial

bias in growth rate for cultured species. Lines connect pairs of measurements for each phylum and are colored by the number of observations in the phylum.

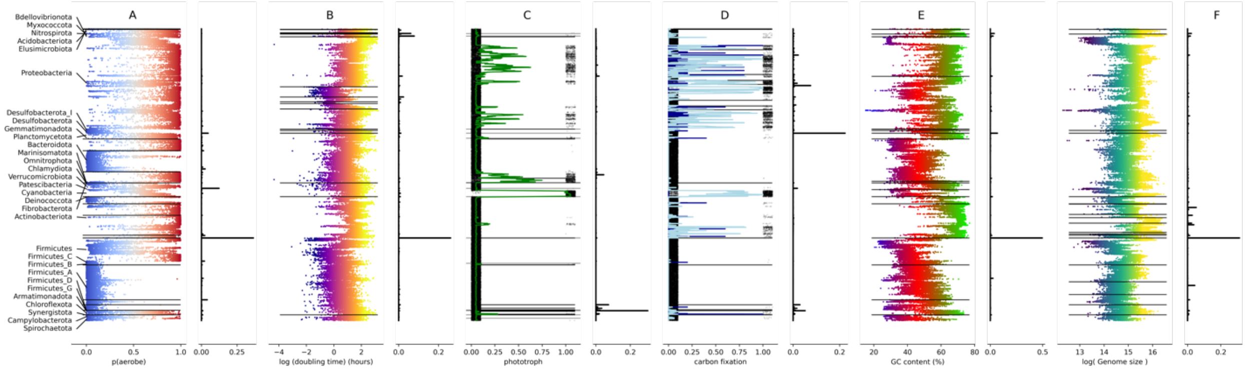

SF7 – Panels A-F show distributions of L2-normalized Haar-like wavelet coefficients for each trait: (A) oxygen tolerance (B) maximum potential growth rate (C) carbon fixation capacity (D) phototrophy (E) GC content and (F) genome size. Panels A-F contain trait values for each genome in postorder in the left plot, and normalized Haar-like wavelet coefficients placed at the y-axis location of the internal node they correspond to in the right plot. Each value in the vector of wavelet coefficients for a trait corresponds to an internal node of the phylogeny, with the wavelet coefficient values describing the amount of variance in a trait uniquely attributed to the differences between the left subtree and the right subtree of the node. Each coefficient is normalized to represent the fraction of the trait's total variance it describes; in a tree with exactly 50% of the species with a trait, and 50% without it, a single coefficient with a magnitude of 1 would be found at the common ancestor of the positive and negative groups. Nodes with larger coefficients have left and right sub-trees that are more different with respect to each trait than nodes with smaller coefficients. Additional lines are drawn over the trait data to mark the edges of the left and right subtrees for the clades with the ten largest wavelet coefficients, illustrating how the wavelet coefficients reflect trait shifts in the data.

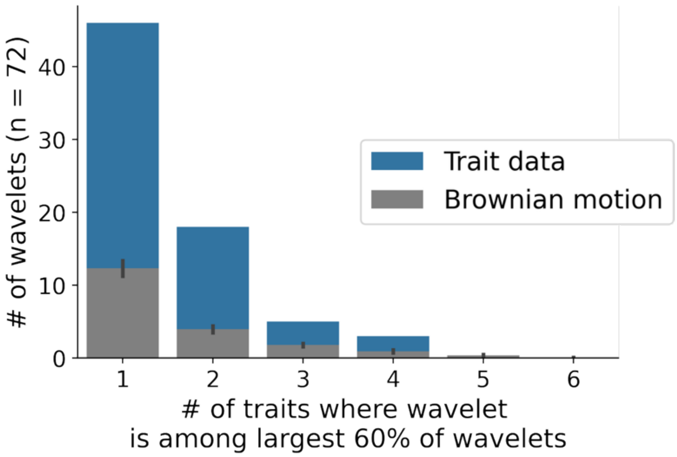

SF8 - After selecting the *n* largest wavelets per trait which accounted for 60% of the total variance in each trait, we found a total of 72 wavelets across all traits. This collection of wavelets

represents the phylogenetic junctions which explain 60% of total trait variance across all six traits. This figure shows the number of traits in which each of these 72 wavelets was among the largest. The data for our six measured traits (blue) is compared against 100 replicates of 6 traits simulated with a Brownian motion model (gray). An identical phylogeny was used to simulate 6 independent traits with $\sigma^2 = 1$, compute the Haar-like wavelet projection, and measure the number of times the same wavelet was among the *n* largest across multiple traits. There were 7.11 ± 1.03 wavelets shared across two, three or four traits across 100 simulations, significantly less than the 26 shared wavelets shared across two or more traits in the actual trait data.

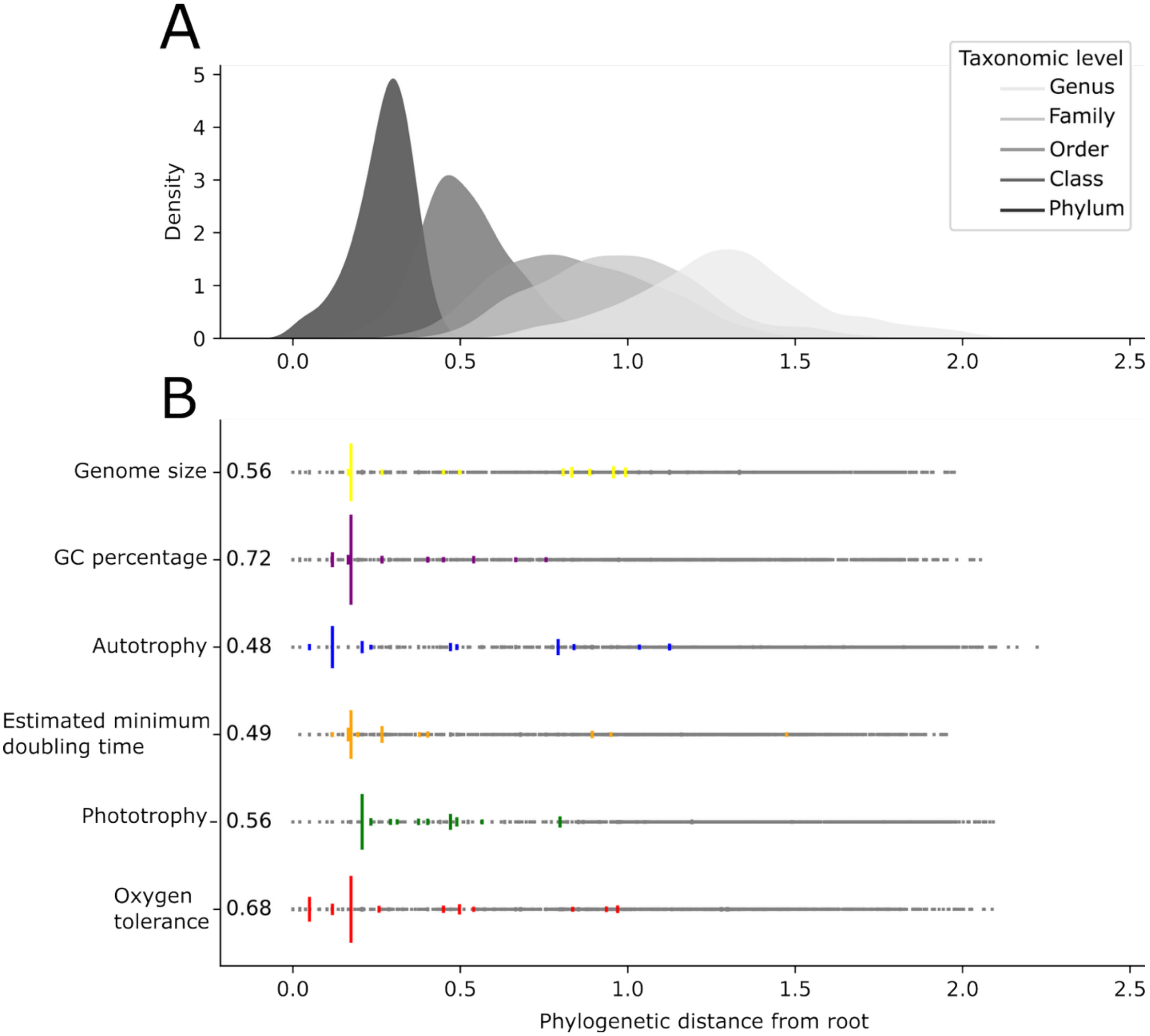

SF9 – The average phylogenetic depth of ancestral nodes (A) and nodes with significant trait variation (B) for each of the six traits, based on the Haar-like wavelet analyses (see Methods). Panel A shows the distribution of root-node distances for the ancestral nodes of each taxonomic level, with shorter distances from the root corresponding generally to more ancient phylogenetic splits and higher taxonomic levels. (B) The phylogenetic distance from the root versus

magnitude of Haar-like wavelet coefficients, a measurement of the amount of trait variance associated with a particular node. The ten largest wavelets are colored for each trait: the total variance captured by the ten largest coefficients is labeled along the y-axis for each trait.

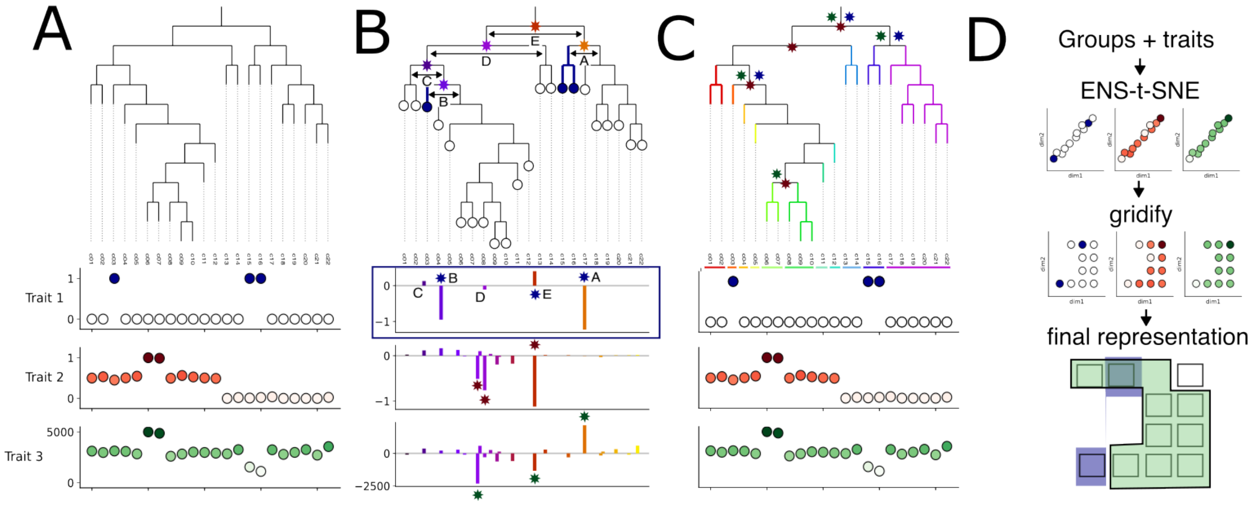

SF10 - Conceptual overview of our approach for generating a periodic table of bacteria. **(A)** Traits with distinct ranges and distributions are associated with tips (species) in a phylogenetic tree. **(B)** Using the Haar-like wavelet transformation, trait distributions are associated with internal nodes using wavelet coefficients, where larger coefficients correspond to nodes with more different left and right subtrees, or nodes which explain more trait variance. Using the example for Trait 1, isolated with a blue box in Panel B: there are 5 non-zero wavelets for Trait 1 because there are five phylogenetic junctions with different trait values in the leaves of their left and right subtrees. The three largest wavelets, corresponding to nodes A, E, and B, are identified. The same process is conducted for each trait; traits with more noise (Trait 3) have larger numbers of nonzero wavelets. The largest wavelets coefficients are identified for each trait and labeled with red, blue, and green starts for Trait 1, Trait 2, and Trait 3 respectively. **(C)** The largest wavelets are used to guide collapsing of the original tree: the nodes corresponding to the largest wavelets for each trait are labeled with stars according to the color scheme in C. Clades which contain no large wavelets define the common ancestor of functionally uniform groups across traits. For example, clade A significantly explains variance in Traits 1 and 3 (blue and green star, rightmost wavelet in Panel B). Because no large wavelets were discovered in the left or right subtree of clade A, its two children are considered "functionally uniform groups." Examining the trait values in in leaves (bottom of panel C) shows this is broadly true, but additional noise could be captured by increasing the number of wavelets considered. **(D)** The rough layout for a "periodic" table is generated by projecting the traits and phylogeny in 2D space with ENS-t-SNE and snapping points to a grid. A final diagram is generated to include additional information on the trait distributions within groups.
